# A Ferritin from *Dendrorhynchus zhejiangensis* with Heavy Metals Detoxification Activity

**DOI:** 10.1371/journal.pone.0051428

**Published:** 2012-12-28

**Authors:** Chenghua Li, Zhen Li, Ye Li, Jun Zhou, Chundan Zhang, Xiurong Su, Taiwu Li

**Affiliations:** 1 School of Marine Sciences, Ningbo University, Ningbo, Zhejiang Province, People's Republic of China; 2 Ningbo City College of Vocational Technology, Ningbo, People's Republic of China; Russian Academy of Sciences, Institute for Biological Instrumentation, Russian Federation

## Abstract

Ferritin, an iron homeostasis protein, has important functions in transition and storage of toxic metal ions. In this study, the full-length cDNA of ferritin was isolated from *Dendrorhynchus zhejiangensis* by cDNA library and RACE approaches. The higher similarity and conserved motifs for ferritin were also identified in worm counterparts, indicating that it belonged to a new member of ferritin family. The temporal expression of worm ferritin in haemocytes was analyzed by RT-PCR, and revealed the ferritin could be induced by Cd^2+^, Pb^2+^ and Fe^2+^. The heavy metal binding activity of recombinant ferritin was further elucidated by atomic force microscopy (AFM). It was observed that the ferritin protein could form a chain of beads with different size against three metals exposure, and the largest one with 35∼40 nm in height was identified in the Cd^2+^ challenge group. Our results indicated that worm ferritin was a promising candidate for heavy metals detoxification.

## Introduction

As one of a major member of iron homeostasis proteins, ferritin plays an important role in storage and detoxification of excess iron in living cells. Structure analysis indicates the protein complex usually composed of 24 subunits, which surrounds an inorganic microcrystalline hollow capable of accommodating up to 4500 Fe^3+^ [1], [2]. In vertebrates, two types of subunits called heavy (H) and light (L) chains are identified and demonstrated to be encoded by separate genes. The H subunit has been studied in a variety of species including vertebrate and invertebrate animals, plants and bacteria [3], [4]. The L subunit, however, has been only found in vertebrates [5], [6]. The H subunits from different species contain seven conserved residues that confer ferroxidase activity for converting Fe^2+^ to Fe^3+^ allowing rapid detoxification of iron cations. The L subunit does not have ferroxidase activity, but serves as a salt bridge that stabilizes the ferritin structure, thus playing a role in iron nucleation and long-term storage [7].

As environmental issue attracted much attention in recent times, development and implication pollutant binding or degradation related genes were considered to be promising way, especially for heavy metals. Accumulative results showed that natural ferritin could store multiple toxic metal ions (Zn^2+^, Pb^2+^, Ni^2+^) by its great storage capacity, resulting to detoxification of heavy metals *in vivo* [8]. Additionally, ferritin had also potential application for chemotherapy and cancer treatment [9].

*Dendrorhynchus zhejiangensis* are one of marine animals belonged to Neatinea, Heteronemertea, Lineidae, *Dendrorhynchus*. The worm lives in the bottom of shrimp ponds, in which the concentration of heavy metal usually higher than that in seawater. Therefore, the worm was considered to be a promising material to study heavy metal detoxification. In the present study, we firstly isolated and characterized a new source of ferritin from *D. zhejiangensis*. Then, its enrichment capacity for different metals *in vitro* was further addressed by atomic force microscopy to fully elucidate the roles of worm ferritin.

## Results

### cDNA library annotation

The QC procedure was performed to evaluate the quality of the cDNA library, and the titer of the cDNA library was 1.7×10^6^ cfu/mL. Colony PCR found that the recombinant rate was 87.5% with average size of 800 bp, indicating that the inserted fragment length was ideal and the library quality was good.

Random sequencing of 458 clones using T3 primer yielded 428 effective sequences. After removing low quality sequences, adaptor and vector sequences, the remaining were subjected for BLASTx analysis with 349 sequences successfully matched. These ESTs were classified into seven categories, including 152 unknown genes, 49 genes related to protein expression, 50 mitochondrial genes, 35 ribosomal structural genes, 19 metabolism-related genes, 11 immune-related genes, and 33 genes related to translation, intercellular material transport, and endocrine.

### Cloning the full-length cDNA of worm ferritin

**Figure 1:**
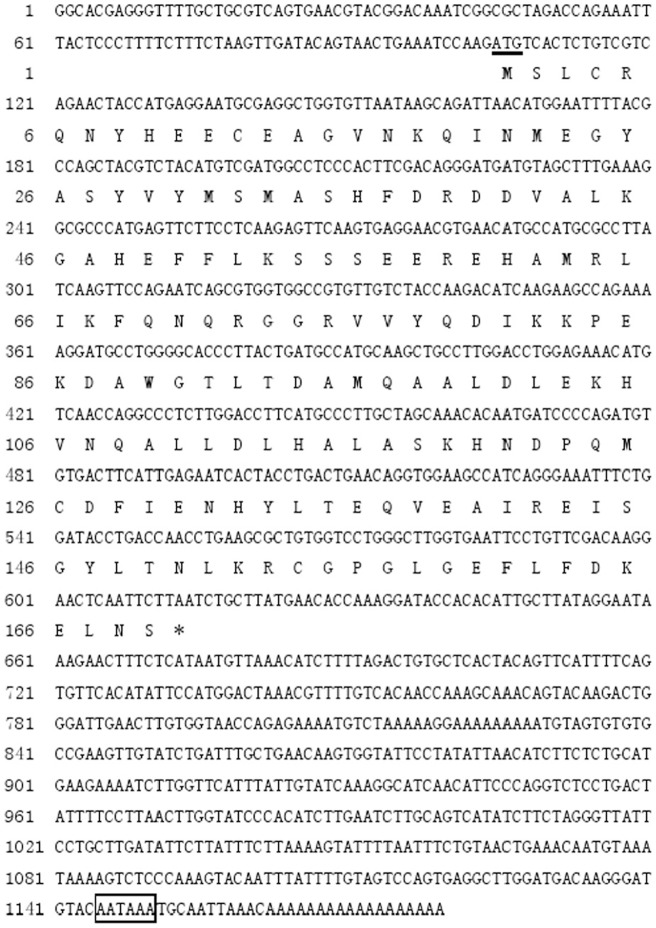
Nucleotide and deduced amino acid sequences of ferritin cDNA of *D. zhejiangensis.* The underlined sequences represent start codons. The polyadenylation signal sequence is shown in box.

**Figure 2:**
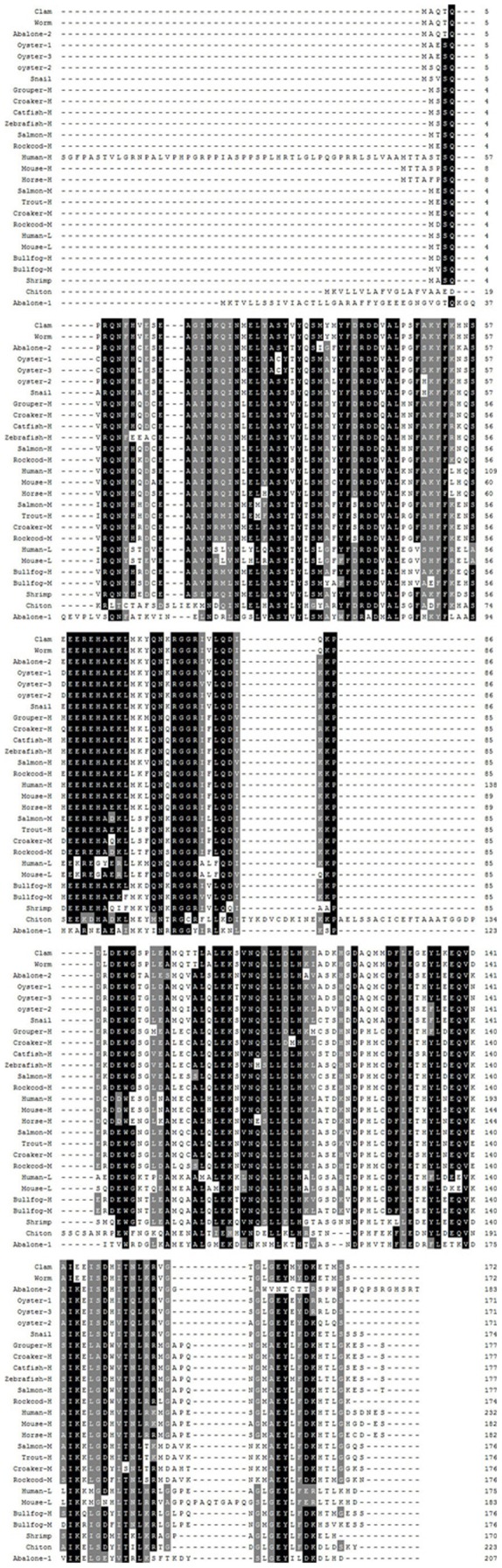
Multiple alignment of worm ferritin with other known ferritins. Amino acid residues that are conserved in at least 80% of sequences are shaded in dark, and similar amino acids are shaded in grey. The detail information for the used sequences were as follows: Human-H (AAH70494), Mouse-H (NP 034369), Horse-H (NP 001093883), Salmon-H (NP 001117129), Salmon-M (ACI67714), Zebrafish-H (NP 571660), Catfish-H (AAY86949), Rockcod-H(P85838), Rockcod-M (P85836), Grouper-H (ABI95136), Trout-H (NP 001118019), Bulkfrog-H (AAA49523), Bulkfrog-M (AAA49525), Croaker-M (ACY75476), Croaker-M (ACY75476), Abalone-1 (ABY87353), Abalone-2 (ACZ732700), Abalone-3 (ABH10672), Abalone-4 (ABG88846), Bay-scallop-1 (ADR71732), Bay-scallop-2 (ADR71731), Crayfish (CAA62186), Fruit-fly (AAF57037), Shrimp-1 (AAX55641), Shrimp-2 (ABB05537), Shrimp-3 (ABP68819), Pearl-shell (ACS72281), Pearl-oyster (AAQ12076), Worm (ACJ37369), Oyster-1 (AAP83794), Oyster-2 (CAD92096), Oyster-3 (CAD91440), Hard-clam (AAZ20754), Pearl-mussel (ADK25061), Snail (AAB24081), Sea-hare (ABF21074), Razor-clam (ACZ65230), Dog-tick (AAQ54712), Winter-tick (AAQ54711), Star-tick (AAQ54708), Sea-cucumber (AAY89589), Oyster-4 (ABE99842), Mussel (ACM86786), Zhikong-scallop (AAV66904) and Crab (ADD17345).

A 1000 bp fragment containing polyA tail was cloned from the *D. zhejiangensis* cDNA library using gene specific primer and T7. Blastx analysis indicated the fragment was similar to the other reported ferritin. The 5′end was obtained with gene specific primer and T3 to get a 750 bp product. By overlapping the three fragments together, an 1179 bp nucleotide sequence representing the full-length cDNA of worm ferritin was assembled. The complete nucleotide and deduced amino acid sequence were shown in Fig. 1. The sequence consists of a 5′-UTR of 104 bp, a 3′-UTR of 565 bp with a poly(A) tail and polyadenylation signal AATAAA, and an ORF of 510 bp encoding a polypeptide of 169 amino acid residues. The three typical ferritin domains for ferritin, ferroxidase diiron center (Glu-23, Tyr-30, Glu-57, Glu-58, His-61, Glu-103, Gln-137), a ferrihydrite nucleation center (Lys-53, Ser-56, Glu-57, Glu-59) and an iron ion channel (His-114, Asp-127, Glu-130), were conserved in worm ferritin (Fig. 2). Blast analysis revealed worm ferritin showed higher simmilarity to other registered counterparts. For example, it shared 71% identities with *Dermacentor variabilis* (AF467696) and *Holothuria glaberrima* (ABS29643), 68% with *Phascolosoma esculenta* (ABW75858), 65% with *Crassostrea ariakensis* (ABE99842), 63% with *Macrobrachium rosenbergii* (ABY75225), and 60% with *Xenopus laevis* (CAA35760) (Fig. 2).

### Transcriptional level expression of ferritin under heavy metals exposure

**Figure 3:**
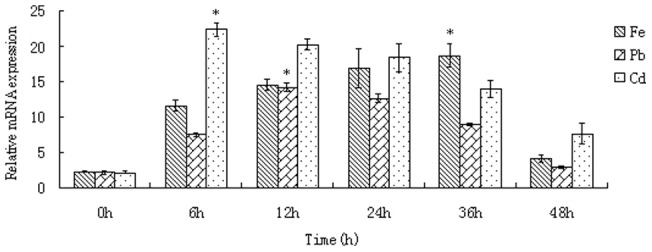
Quantification of worm ferritin mRNA expression under Fe^2+^, Pb^2+^ and Cd^2+^ exposure by qPCR.

Temporal effect of heavy metals exposure on the transcriptional activities of worm ferritin were investigated by qPCR (Fig. 3). In Fe^2+^ groups, an increase expression profile was detected at the first 36 h, and the peak expression was reached at 36 h with 18.5-fold increase compared to the control group (P<0.05). In Pb^2+^ group, the ferritin mRNA level increased and reached maximal level at 12 h with 7-fold increase compared to the control group (P<0.05). In Cd^2+^ group, the ferritin mRNA expression was sharply induced during the first 6 h, then slowly decreased with time going on (Fig. 3).

### Recombiant expression of worm ferritin

**Figure 4:**
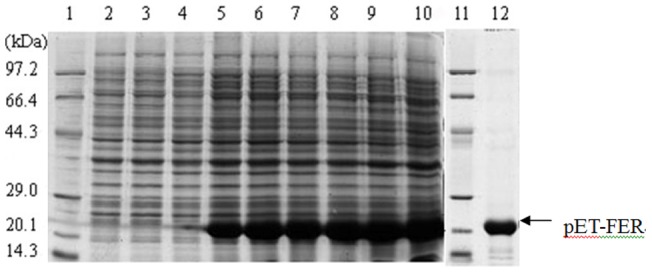
Ferritin expression and purification revealed by SDS-PAGE. lane 1 and11: low molecular marker; lane 2, 3 and 4: negative control; lane 5–10: induce expression at 1 h, 2 h, 3 h, 4 h,5 h and 6 h; lane 12: purified expression product.

The positive transformants were analyzed by SDS-PAGE to characterize the recombinant product of worm ferritin. After IPTG induction, an obvious protein band with molecular weight between 20.1 kDa and 29 kDa was detected in the positive transformants (Fig. 4), which could be purified to homogeneity by HiTrap Chelating Columns. The molecular mass of the purified product was in good agreement with the Predicted MW of worm ferritin. Moreover, the intensity of the recombinant protein band was increased with time went on. The peak expression level of recombinant protein was observed at 6 h after IPTG was introduced into the culture.

### Wstern blot analysis of worm ferritin expression

**Figure 5:**
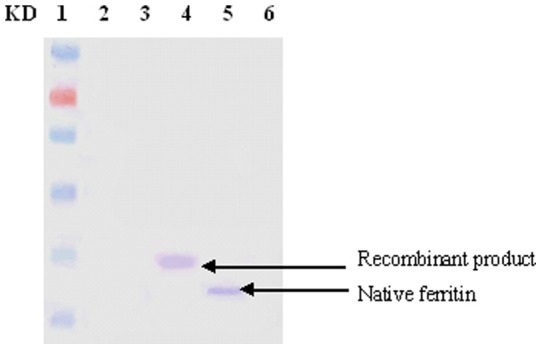
Specificity of worm ferritin polyclonal antibody determined by Western-blot. Lane 1: protein marker; lane 2 and 3: negative control; lane 4: recombinant product; lane 5: total protein extraction from worm; lane 6: antiserum control.

With the purified recombinant worm ferritin, polyclonal antibodies were generated for Western blot (Fig. 5). The results showed that antiserum could be specifically identified by not only the recombinant protein, but also the native protein from haemocytes. No signals were detected in other control samples. The size difference between the pET-FER and native ferritin was contributed to the extra fusion expression tag in the pET28 vector.

### Characterization of recombiant worm ferritin binding by atomic force microscopy

**Figure 6:**
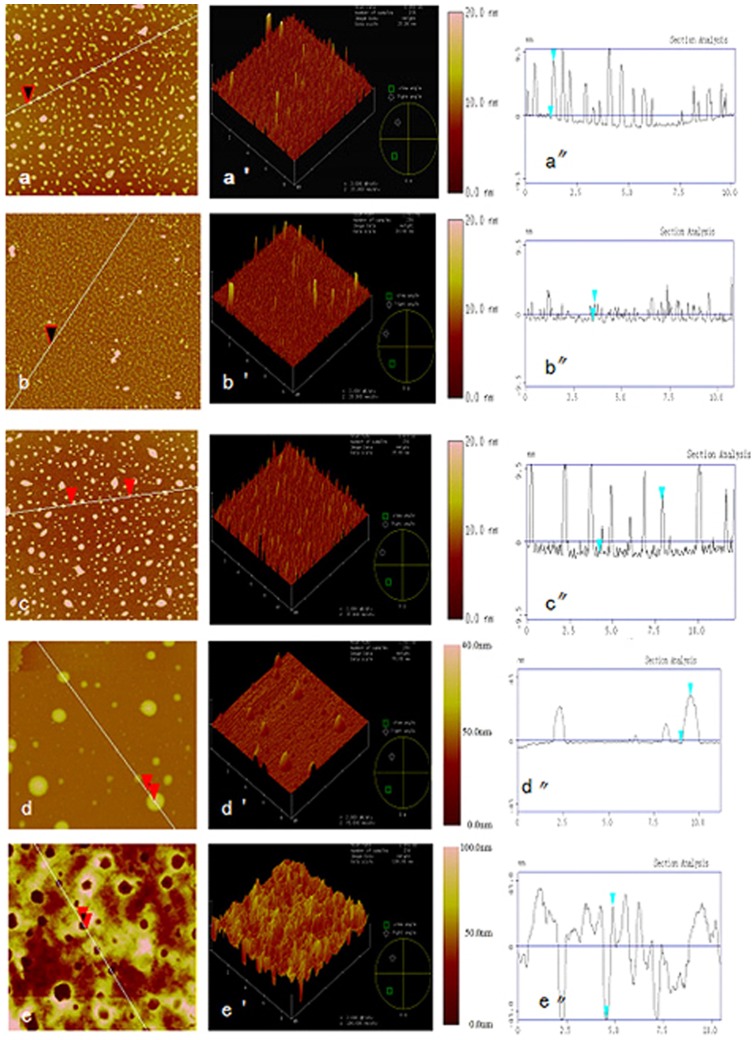
Interaction between recombiant ferritin and metal ions by AFM with 9×9 **μm^2^ scanning range.** a: Ferritin training complex topography image of the front surface, a’: three-dimensional topography map from the a, a”: relative height of the peak figure from the a; b: Ferritin culture surface topography map refolded, b’: three-dimensional topography map from the b, b’’: Relative height of the peak figure from the b; c: Incubation of ferritin and Fe^2+^ at 37°C for 30 min, c’: three-dimensional topography map from the c, c’’: Relative height of the peak figure from the c; d: Incubation of ferritin and Cu^2+^, at 37°Cfor 30 min a, d’: three-dimensional topography map from the c, d’’: Relative height of the peak figure from the d; e: Incubation of ferritin and Cd^2+^ at 37°C for 30 min, e’: three-dimensional topography map from the e, e’’: Relative height of the peak figure from the e;.

The majority of ferritin molecules were well dispersed and had height of 6∼12 nm in cross-section analysis before refolding (Fig 6aa”). In addition to a small amount of protein aggregates, most of the ferritin molecules formed a chain of beads with height of 5∼10 nm and diameter of about 90∼100 nm (Fig. 6bb”). After adding Fe^2+^, Pb^2+^, and Cd^2+^, respectively, ferritin molecules aggregated as protein complexes with height of 9∼13 nm and diameter of about 300∼700 nm (Fig 6cc”), height of 35∼40 nm and diameter of 400∼1000 nm (Fig. 6dd”), and height of 95∼100 nm and diameter of about 400∼1100 nm (Fig. 6ee”), respectively.

## Discussion

Atomic force microscopy (AFM) has become an important tool for studying the interaction between biological molecules based on its higher resolution and capability. The technique had been suceessfully utilized in antigen and antibody, DNA and protein complex, and DNA conformation [10]. In this study, a full-length cDNA of worm ferritin was firstly cloned by cDNA library and RACE, then, its expression profiles were characterized at mRNA level under heavy metal exposure. Its binding activity to heavy metals was further investigated by atomic force microscopy (AFM).

In order to better understand the role of the worm ferritin in response to heavy metals challenge, temporal expression levels of worm ferritin were analyzed by real-time PCR. We showed that the expression of the ferritin in all treated groups was significantly increased after 6 h of exposure to three heavy metals, indicating that worm ferritin could be induced by Cd^2+^, Pb^2+^ and Fe^2+^. The ferritin expression level of Cd^2+^ at same dose was about three times higher than that of Pb^2+^, suggesting that worm ferritin may be the least sensitive to Pb^2+^, compared with Cd^2+^ and Fe^2+^. The similar patterns of ferritin toward heavy metals exposure was also reported in giant prawn [11], clam [12], fruit fly [13] and amphioxus [14]. Given these facts, our results suggested that the enhanced expression of worm ferritin by heavy metals treatments was probably a protective mechanism of the cell to environmental stress [15].

In order to utilize the gene in practice, knowledge on how worm ferritin binding with heavy metals should be further investigated. The dynamic process between worm ferritin and three heavy metals was observed with AFM. The size of recombinant ferritin was very similar to that of the native ferritin protein after refolding [16], with small amount of irregularly aggregated molecules and fragments. However, when divalent ions was introduced in the culture, the subunit number of ferritin shell gradually increases, and ferritin shell becomes thicker, resulting in the size of ferritin structure was greater than that of native ferritin [3]. The interactions between metal and its binding ligands, such as hydrophobic bonds, hydrogen bonds, electrostatic interaction and van der Waals force are changed, which significantly impacts protein folding. Harrison et al [17] classified ferritin's iron storage procedure into three stages, namely, the oxidation of Fe^2+^ to Fe^3+^, movement of Fe^3+^ and the nucleation of iron core. Once the small organic molecules occupied part of the iron core, unstable iron ions in ferritin were released, resulting in decrease of electron density within the iron core [18]. As a continuation of storage reaction, the amount of released iron increased. The three-phase material exchange tunnel was narrowed by ferrtin shell through a flexible regulation. Thus, the width of ferritin was decreased. Our result was in agreement with the theory that free or denatured ferritin subunits could quickly wrap bare iron core to form native structure.

Regarding to Cd^2+^ challenge, the dramatic conformation changes of ferritin was also observed through forming a large hollow, which was speculated to be a cadmium core formed by a lot of Cd^2+^ gathering and surrounded by many ferritin molecules. Previous reports had shown Cd^2+^ was capable of binding to both the inside and outside of horse spleen ferritin [19]. All these evidences indicated worm ferritin might be promising candidates for Fe^2+^ and Cd^2+^ detoxification.

The introduce of heavy metal into recombinant ferritin was also changed its size. The diameter of ferritin increased from 35∼40 nm to 400∼1000 nm when added with Pb^2+^. It was hypothesized that the hydration of metal ions destroyed the hydration layer surrounding ferritin molecules, making it easier to form folding intermediates. The stronger the metal ion hydration was, the greater role it played in promoting ferritin aggregation, leading to the formation of large ferritin aggregates. Therefore, metals with large electron could be more likely to promote ferritin aggregation because they had higher hydration free energy [20]. In addition to metal hydration, the electrostatic interaction among metal ions shielded electrostatic repulsion among protein molecules, the so-called Debye screening. In alkaline solution, ferritin molecules had negative charges, thus the electrostatic repulsion among them made aggregation difficult. These phnomena was also expained by the fact that metal ions could accelerate protein nucleation. Metal ions with high positive charges exerted much stronger Debye screening effect. Therefore, they were more likely to enhance nucleation than metal ions with lower positive charges. Nucleation rate could also affect the protein shape, thus exposure to different metal ions had the potential to form different protein shapes [21].

In summary, we cloned the full-length cDNA of *D. zhejiangensis* ferritin and characterized its expression patterns during exposure to iron ion. Subsequently, the recombinant ferritin was produced to investigate its binding activity to Fe^2+^, Pb^2+^, and Cd^2+^ by AFM. All our results supported that worm ferritin was a promising candidate for heavy metals detoxification. In order to utilize the protein in practice, its binding capcity to different heavy metals should be elucidated in our further work.

## Materials and Methods

### Worms

*Dendrorhynchus zhejiangensis* was collected from shrimp ponds along the coast of Hudoudu (29.6° N, 121.6° E), Fenghua City, Zhejiang Province, China in May 2011. The animals were acclimated in our laboratory for two days prior to the experiment. No specific permits were required for the described field studied, and no specific permissions were required for these locations.

### cDNA library construction and EST analysis

Eight *D. zhejiangensis* were randomly collected and homogenized with liquid nitrogen. The lysate was used for cDNA library construction. The cDNA library was constructed using the ZAP-cDNA synthesis kit and ZAP-cDNA GigapackIII Gold cloning kit (Stratagene). Random sequencing of the library using T3 primer yielded 458 successful sequencing reactions. BLAST analyses of all the 458 EST sequences revealed that one EST of 387 bp was highly similar to the previously identified ferritin. Therefore, the EST sequence was selected for the full-length cDNA cloning.

### Cloning the full-length cDNA of worm ferritin

Two specific primers, Fer-R: 5′-GTGGAAGCCATCAGGGAA-3′ and Fer-F: 5′-AAGGGCATGAAGGTCCAAGAGGG-3′, were designed based on the EST to amplify the 3′ and 5′ ends of worm ferritin combined with vector prime T7 or T3, respectively. PCR was performed at 94°C for 5 min followed by 35 cycles of 94°C for 30 s, 58°C for 30 s, and 72°C for 1 min and final extension at 72°C for 10 min. The obtained PCR amplicons were gel-purified and cloned into pMD18-T (Takara, China). After transforming into competent *E. coli*, the positive colonies were identified by colony PCR. Three positive clones were sequenced at Invitrogen (Shanghai, China) and were analyzed using BLAST (http://www.ncbi.nlm.nih.Gov/BLAST/).

### Quantitative expression analysis of ferritin

For heavy metals challenge experiment, worms were divided in to four tanks and were exposed to three metals of Fe^2+^, Pb^2+^ and Cd^2+^ with the final concentration of 10 μM. The fourth tank was served as control group. The seawater was changed daily and metal stock solution was added to the seawater everyday. After 6 h, 12 h, 24 h and 48 h exposure, the haemocytes from each group were collected from the control and the treated groups for RNA extraction and cDNA synthesis.

For expression analysis, the ferritin specific primers, FER-F (5′-GAGGAATGCGAGGCTGGT-3′) and FER-R (5′-CACG CTGATTCTGGAACTTG-3′), were designed to amplify a product of 205 bp. For normalizing the ferritin transcripts, two β-actin specific primers actin-F (5′-GGACCTCTACGCCAACACTG-3′) and actin-R (5′- ATGCAAGGATGGAGCCAC-3′) were used to amplify an 170 bp fragment. The fluorescent real-time PCR assay was carried out performed in Rotor-Gene 6000real-time PCR detection system and the condition was according to our previous work [12]. The 2^−ΔΔCT^ method was used to analyze the expression level of worm ferritin. All data were given in terms of relative mRNA expressed as mean ± S.D. The data were then subjected to analysis by one-way analysis of variance (ANOVA). Differences were considered significant at *P*<0.05.

### Expression and purification of worm ferritin

PCR product corresponding to ferritin ORF was amplified by two gene specific primers with *BamH* I and *Hind* III sites at 5′ends. The PCR amplicons were digested with *BamH* I and *Hind* III, and then inserted into digested pET-28a(+) vector. After transforming into competent *E. coli* BL21, positive clones were identified by kanamycin screening and sequencing. After sequencing to ensure in-frame insertion, positive clones were incubated at 37°C at 220 rpm. When the culture reached OD_600 nm_  = 0.5 to 0.7, 1 mM isopropyl-β-D-thiogalacto- pyranoside (IPTG) were added to induce the ferritin expression for additional 6 h. Cells were harvested at 1 h, 2 h, 3 h, 4 h, 5 h and 6 h by centrifugation at 4000 r/min for 15 min and resuspended in PBS for sonication. The expressed ferritin was purified using Ni-NTA affinity column (GE healthcare) according to the manufacturer's instruction.The expression and purified products were separated by SDS-PAGE and stained by Coomassie brilliant G250.

### Western blot analysis

Protein samples from positive bacteria and *D. zhejiangensis* tissue were separated by SDS-PAGE. Proteins were electronically transferred onto nitrocellulose membrane, and blocked with 5% non-fat milk in TBST. Then the membrane was incubated with anti-ferritin serum for 1 h and washed three times with TBST. The membrane was then incubated with 1∶10000 diluted rabbit anti-mouse secondary antibody for 1 h and washed three times with TBST. Signals were visualized using NBT/BCIP method and the reaction was terminated with distilled water. The anti-ferritin serum was prepared according to our previous work [4].

### Functional characterization of worm ferritin

To achieve recombinant ferritin with biological activity, the purified protein was refolded against stepwise decrease of urea concentration in GSH/GSSG buffer (50 mM Tris–HCl, 1 mM EDTA, 50 mM NaCl, 10% glycerol, 1% glycine, 2 Mm reduced glutathione, 0.2 Mm oxide glutathione, pH 8.0) overnight at 4°C. The concentration of purified protein was quantified by BCA method.

To determine heavy metal enrichment capacity, 1 mL purified ferritin (20 μg/mL) was incubated with 20 μL of 2 mM FeCl_2_, PbCl_2_ and CdCl_2_ at 22°C for 10 min, respectively. Then, 5 μL of each mixture was evenly distributed on the surface of pretreated mica sheet and dried for 3 h at room temperature at environmental humidity of 5%. The samples were observed with a Nanoscope IIIa multimode scanning probe microscopy (Veeco Instruments, USA) using Tapping mode. The NSC11 triangular cantilever probe (MikroMasch, USA) was used for scanning at a resonance frequency of 330 kHz, scan rate of 1–1.5 Hz under spring constant of 50 N/m.
